# Aflatoxin Exposure from Milk in Rural Kenya and the Contribution to the Risk of Liver Cancer

**DOI:** 10.3390/toxins11080469

**Published:** 2019-08-10

**Authors:** Anima J. Sirma, Kohei Makita, Delia Grace Randolph, Daniel Senerwa, Johanna F. Lindahl

**Affiliations:** 1 State Department of Livestock, Ministry of Agriculture, Livestock, Fisheries and Irrigation Kenya P.O Private bag Kangemi, Nairobi 00100, Kenya; 2 Department of Veterinary Medicine, School of Veterinary Medicine, Rakuno Gakuen University, 582 Bunkyodai Midorimachi, Ebetsu 069-8501, Japan; 3 Department of Biosciences, International Livestock Research Institute, P.O. Box 30709, Nairobi 00100, Kenya; 4 Department of Public Health and Toxicology, University of Nairobi, P.O. Box 29053, Nairobi 00625, Kenya; 5 Department of Clinical Sciences, Swedish University of Agricultural Sciences, P.O. Box 7054, SE-750 07 Uppsala, Sweden; 6 Department of Medical Biochemistry and Microbiology, Uppsala University, P.O. Box 582, 75123 Uppsala, Sweden

**Keywords:** mycotoxins, risk assessment, food safety standards, hepatocellular carcinoma, East Africa

## Abstract

Milk is an important commodity in Kenya; the country has the largest dairy herd and highest per capita milk consumption in East Africa. As such, hazards in milk are of concern. Aflatoxin M_1_ (AFM_1_) is a toxic metabolite of aflatoxin B_1_ (AFB_1_) excreted in milk by lactating animals after ingesting AFB_1_-contaminated feeds. This metabolite is injurious to human health, but there is little information on the risk to human health posed by AFM_1_ in milk in rural Kenya. To fill this gap, a quantitative risk assessment (QRA) applying probabilistic statistical tools to quantify risks was conducted. This assessed the risk of liver cancer posed by AFM_1_ in milk, assuming 10-fold lower carcinogenicity than AFB_1_. Data from four agro–ecological zones in Kenya (semi-arid, temperate, sub-humid and humid) were used. We estimated that people were exposed to between 0.3 and 1 ng AFM_1_ per kg body weight per day through the consumption of milk. The annual incidence rates of cancer attributed to the consumption of AFM_1_ in milk were 3.5 × 10^−3^ (95% CI: 3 × 10^−3^–3.9 × 10^−3^), 2.9 × 10^−3^ (95% CI: 2.5 × 10^−3^–3.3 × 10^−3^), 1.4 × 10^−3^ (95% CI: 1.2 × 10^−3^–1.5 × 10^−3^) and 2.7 × 10^−3^ (95% CI: 2.3 × 10^−3^–3 × 10^−3^) cancers per 100,000 in adult females, adult males, children 6–18 years old, and in children less than five years old, respectively. Our results show that aflatoxin exposure from milk contributes relatively little to the incidence of liver cancer. Nonetheless, risk managers should take action based on cumulative exposure from all sources of aflatoxins.

## 1. Introduction

Worldwide, most countries have set standards for aflatoxins in food and feed in order to protect their markets and safeguard people from the harmful health effects of aflatoxins. Human dietary exposure in Kenya is evident from past outbreaks of aflatoxicosis [1]. Foods susceptible to aflatoxin contamination include maize, groundnuts, millet, sorghum and dairy products. Aflatoxin M_1_ (AFM_1_) is a metabolite of aflatoxin B_1_ (AFB_1_) encountered in milk and milk products from animals exposed to AFB_1_-contaminated feeds [2]. Aflatoxin has been reported to occur in these at levels above the Kenyan regulatory limits of 5 µg/kg AFB_1_ in foods/feeds (levels for dairy are not specified) and the European Union (EU) recommended limits of 50 ng/kg AFM_1_ in dairy [3,4,5,6,7]. Aflatoxin-contaminated foods in Kenya reach consumers easily through informal markets, where most produce is sold and where regulatory enforcement is inadequate.

Aflatoxins, produced by the *Aspergillus* species, are poisonous fungal by-products that cause health effects that vary from acute to chronic illness, notably cancer. The International Agency for Research on Cancer (IARC) classifies naturally occurring aflatoxins as Class 1 carcinogens, meaning that they have been confirmed to cause liver cancer in most animal species studied and in humans [8,9]. Liver cancer is ranked as the fourth leading cause of cancer death worldwide and is much more common in men than in women. The standardized incidence rate is 15.3 per 100,000 among men and 5.4 per 100,000 among women [10]. In the USA, the incidence of primary malignant liver cancers is around 1 per 100 million children [11]. In 2012, an estimated 1120 new liver cancer cases and 1037 liver cancer deaths occurred in Kenya [12]. Hepatocellular carcinoma (HCC) is the most common primary liver cancer worldwide. Risk factors for HCC include hepatitis B virus (HBV) infection, hepatitis C virus (HCV) infection, aflatoxicosis, alcoholism, smoking, and hereditary conditions [13]. HCC rates are particularly high in eastern/south-eastern Asia and Africa, where concurrent infections with hepatitis virus and aflatoxin exposure increase the risk of developing the disease. Each year, it is estimated that between 14% and 19% of new HCC cases worldwide could be attributed to aflatoxin exposure [14]. 

**Figure 1:**
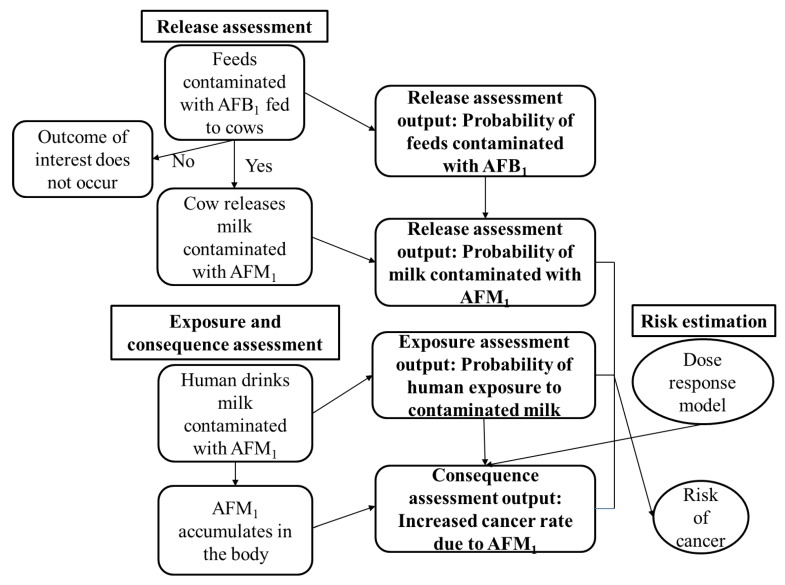
A scenario tree for estimation of risk for liver cancer for humans following consumption of AFM_1_ contaminated milk.

In order to quantify the risk of HCC in children and adults exposed to aflatoxins, a risk assessment can be performed. A risk assessment is the assessment, either qualitative or quantitative, of the probability and the impact of a hazard (risk is the combination of probability and impact) [15]. Risk assessment is usually based on the World Organization for Animal Health (OIE) or the Codex Alimentarius Commission framework [16,17]. Risk assessment should guide food regulators and scientists in undertaking risk management processes, such as the setting of legislative levels or guideline maximum levels for mycotoxins in food supplies [18]. Shephard [19] provided an estimate for the population risk for HCC from aflatoxin B_1_ (AFB_1_) exposure in maize from urban and rural markets in Kenya at 11 and 29 cancers/year per 100,000 population, respectively. Similar estimates for risk of liver cancer from consumption of aflatoxin M_1_ (AFM_1_) in milk from Kenya have not been made. Furthermore, the Kenyan government has not set official limits for AFM_1_ in milk [20], but it frequently refers to the EU standards, which are 50 ng/kg [21]. The carcinogenic potency for AFM_1_ has been calculated to be 10% of the potency of AFB_1_ based on the induction of HCC in AFB_1_-treated rats versus AFM_1_-treated rats [22]. A survey of occurrences of AFM_1_ in marketed milk in Nairobi city, Kenya found that more than 50% of samples were contaminated at levels above the EU limits of 50 ng/kg [23]. A separate survey of raw milk samples from rural Kenya found that more than 10% of milk samples were contaminated above the EU 50 ng/kg limits [6]. The high occurrence of aflatoxins in milk in Kenya warrants the need for quantifying its health risks. In this paper we present a quantitative risk assessment to estimate aflatoxin exposure from cow’s raw milk in rural Kenya and its contribution to the risk of HCC, using the scenario tree depicted in Figure 1.

## 2. Results

### 2.1. Release Assessment

In total, 512 milk and 144 feed samples were analyzed for aflatoxins using ELISA. Overall, 73% of the feeds were above Kenyan limits of 5 µg/kg AFB_I_. Ten per cent of the milk samples exceeded the EU limits of 50 ng/kg AFM_1_. Samples from the humid agro–ecological zone (AEZ) were most likely to exceed the EU limits (*p* < 0.05). A comparison of AFM_1_ levels in milk from cows fed with or without concentrates/maize-based feeds is shown in Table 1. 

### 2.2. Exposure Assessment

On average, 0.4 liters of milk were consumed each day across the agro–ecological zones (AEZs). The temperate region had the highest average milk consumption compared to the rest of the AEZs (*p* < 0.01). No significant difference was observed in average milk consumption among the humid, sub-humid and semi-arid AEZs (*p* > 0.05; Table 2).

AFM_1_ exposure through milk ranged from 0.3 to 1 ng/kg of body weight per day. Children less than five years old had the highest exposure estimate at 1 (95% CI: 0.6–1.4) ng/kg of body weight per day. Adult females followed at 0.4 (95% CI: 0.2–0.5) ng/kg of body weight per day. Adult males and children aged 6–18 years old both had a mean exposure estimate of 0.3 (95% CI: 0.1–0.5) ng/kg of body weight per day.

### 2.3. Consequence Assessment and Risk Estimation

The probability distributions of risk of cancer across the various AEZs disaggregated by gender are shown in Figure 2, Figure 3, Figure 4 and Figure 5 and described below. Shown in the supplementary material are Figures S1–S16, the probability distributions for adult females, adult males, children 6–18 years, and children up to five years, in each AEZ, respectively. 

Among adult males, the annual incidence rate was estimated at 2.9 × 10^−3^ (95% CI: 2.5 × 10^−3^–3.3 × 10^−3^) cancers per 100,000; 3.5 × 10^−3^ (95% CI: 3 × 10^−3^–3.9 × 10^−3^) cancers/year per 100,000 in the adult female category; 1.4 × 10^−3^ (95% CI: 1.2 × 10^−3^–1.5 × 10^−3^) cancers/year per 100,000 among children aged 6–18 years old; and 2.7 × 10^−3^ (95% CI: 2.3 × 10^−3^–3 × 10^−3^) cancers per year per 100,000 among children less than five years old. Most categories from the humid AEZ had higher annual incidence rates than the other AEZs (Table 3).

## 3. Discussion

Risk assessment is the process of estimating the magnitude and the probability of a harmful effect on individuals or populations from specified agents or activities [24]. In this study, we conducted a quantitative risk assessment of hepatocellular carcinoma (HCC), taking into consideration hepatitis B virus prevalence in Kenyan rural populations and aflatoxin exposure through milk in four AEZs. Milk is consumed by most Kenyan adults and children, mainly boiled in tea and porridge [25]. The boiling or pasteurization of milk does not remove aflatoxins, as they are heat stable [26]. Generally, the amount of AFM_1_ excreted into milk varies from less than 1% to 7% of the dose of AFB_1_ ingested by the cow [27,28]. In this study, 73% of the feeds had AFB_1_ beyond Kenyan regulatory limits. This is consistent with levels reported in feeds from urban centers in Kenya [29]. Milk from cows fed concentrates or maize-based feeds had higher AFM_1_ levels compared to those not fed. This is because commercial feeds are much more likely to be contaminated with aflatoxins than hay or fodder stored at the farms [30]. AFM_1_ exposure assessment showed a range of between 0.3 and 1 ng/kg of body weight, which is similar to the amount reported in Argentina of 1.22 ng/kg body weight [31]. 

Globally the standardized annual incidence rate for liver cancer is 15.3 per 100,000 among men and 5.4 per 100,000 among women [8]. In this study, based on the levels of AFM_1_ and the consumption of milk in rural Kenya, and assuming a 10-fold lower carcinogenicity than AFB_1_, the calculated annual incidence rate was 0.00294 and 0.00347 per 100,000 in males and females, respectively. These latter figures contribute 0.02% and 0.06% to the global incidence rates. This thus shows that AFM_1_ from milk consumption contributes very little to the annual incidence rate for liver cancer. In general, the incidence rates for HCC reported here are comparable to estimates for Gambia [18] but relatively lower than those reported from a risk assessment in Kenya based on aflatoxin exposure from groundnuts [32] and maize [18]. The latter study reported an incidence rate of 29.2 and 11 cancers per year per 100,000 population from maize collected from rural markets and commercial markets, respectively. The relatively higher incidence rates reported are likely due to a focus on maize, in which much higher aflatoxins levels are reported compared to milk in Kenya, and the higher carcinogenicity of AFB_1_. In addition, that study assumed a higher prevalence of hepatitis B (25%) than the 13% that was used in this study. Liver cancer is the fifth most common cancer in men and the ninth most common cancer in women, and it is largely a problem of the less developed regions [12]. This assessment found almost matching annual HCC incidence rates for both females and males which may indicate that exposure to AFM_1_ through milk occurs equally in both genders. However, this study did not consider other possible differences, such as different HBV prevalence in men and women, exposure to other carcinogens such as alcohol, or risk factors such as obesity. Estimates for children did not take into account different base rates in this population, as there was no information on this for Kenya.

Higher annual HCC incidence in the humid AEZ is consistent with more AFM_1_ contamination beyond the EU limits in milk in these areas. The same AEZ recorded high aflatoxin contamination levels in feeds and other susceptible foods of maize, millet and sorghum [6,7]. High humidity supports mold growth in foods and feeds and possible aflatoxin production. The control of aflatoxins in dairy feeds would significantly reduce the carryover of aflatoxins to milk and other animal products intended for human consumption. Key interventions to reduce aflatoxins in animal feeds include keeping moisture and temperature of feeds moderately low (<13%) to inhibit mold growth, keeping equipment used on-farm clean, and, where possible, using mold inhibitors or binders [30].

This study calculated the risk of HCC by assuming that the carcinogenicity of AFM_1_ is 10 times lower than of AFB_1_. This is based on rather weak evidence from animal trials: If carcinogenicity is higher in humans, then the relative contribution of AFM_1_ would be higher. Moreover, concerns over aflatoxin in milk are not only related to cancer cases but also to the risks of stunting and immunosuppression in young children. Thus, there may be more risks with AFM_1_ in milk products than shown by this risk assessment. The risk assessment method follows the OIE method as opposed to the Codex Alimentarius risk assessment, which is suitable for microbiological risks [17]. Another method is the margin of exposure approach, which has been used successfully for other dietary carcinogens [33,34], but this requires more information on benchmarking doses. However, it is unlikely that another method would have given final estimates of completely different magnitudes. While the risk assessment here was based on data from rural Kenyan farmers, the estimate of the risk was of the same magnitude as the estimates done by Ahlberg et al. [35] for urban populations. 

## 4. Conclusions

In conclusion, we demonstrate for the first time that AFM_1_ is likely to contribute to a small proportion of HCC cases occurring in rural Kenya. Despite the relatively low annual HCC incidence rates from exposure through milk, there is still reason for risk managers to take action due to the cumulative exposure from all sources of aflatoxins. In addition, the prognosis for liver cancer is very poor, with an overall ratio of mortality to incidence of 0.95 [12]. We hope that the risk estimates provided here will guide the Kenyan authorities in setting legislative levels for AFM_1_ in milk and milk products.

## 5. Materials and Methods

### 5.1. Study Site and Household Selection

This study was based on data from a cross-sectional study in 2015 in five counties representing four agro–ecological zones (AEZs) in Kenya. The counties selected were: Isiolo (semi-arid), Tharaka-Nithi (humid), Kwale (sub-humid), Bungoma (temperate), and Kisii (temperate). 

The household size calculation and sampling has been described elsewhere [6,7]. Briefly, the number of households sampled were calculated based on an expected aflatoxin occurrence of 72% at the 95% confidence level with a 10% desired level of precision [36]. The calculated sample size was 321 dairy cattle farms, which were divided equally to the five counties resulting in a sample size of 64 farmers per county.

### 5.2. Aflatoxin Determination and Validation Of Method

The levels of AFM_1_ used for the assessment of risk of cancer in this paper were analyzed using a commercial competitive enzyme-linked immunosorbent assay (ELISA) method described by Senerwa et al. [6]. Tests were performed according to manufacturer’s instructions. The given limit of detection of the test was 2 ng/kg. To evaluate the accuracy, precision, and linearity of each ELISA plate reading, a calibration curve was made from calculated values of standards provided by the manufacturer. The standards had concentrations of 0, 5, 10, 25, 50 and 100 ng/kg. A regression coefficient (r^2^) was calculated from the calibration curve. ELISA plate readings with a regression coefficient (r^2^) less than 0.95 were repeated. With regards to validity of the data, Imtiaz and Yunus [37] reported recovery data from milk of between 94%–115%. For animal feeds, two samples each from categories of low (0–5 µg/kg), medium (5.1–20 µg/kg) and high levels (20.1–10,000 µg/kg) were tested using a Shimadzu Nexera X2 ultra performance liquid chromatograph (UPLC) fitted with a prominence fluorescence detector (RF-20A XS). Correlations tests were done to check for the agreement of results. Included in the validation sample were two known concentrations (5 and 32 µg/kg) of certified corn reference material obtained from the Office of the Texas State Chemist. UPLC readings whose determined aflatoxin concentration of the reference material were off the range were repeated. 

### 5.3. Quantitative Risk Assessment

A quantitative risk assessment (QRA) was performed based on the OIE framework comprised of four steps, namely release assessment, exposure assessment, consequence assessment, and risk estimation [17]. A QRA model was developed by incorporating the four steps in Microsoft Office Excel with @RISK software version 6.0 (Palisade Corp, Ithaca, NY, USA) included as an add-in. A schematic presentation of the model is shown in Figure 1. The @RISK software was used to analyze the QRA data. The data used in each QRA step are described below, and a summary is shown in Table 4. The data were disaggregated by agro–ecological zones, gender, and age groups in order to understand the health risks of sub-populations among rural farming households.

#### 5.3.1. Release Assessment 

Release assessment involves the description of the biological pathways necessary for an activity to ‘release’ pathogenic agents into a particular environment. The release assessment was based on the following data: Type of farming (either intensive or extensive), feeding of maize-based feeds, number of farms, lactating animals on concentrates and those without, total milk produced, and levels and occurrence of aflatoxins in feeds and milk. The apparent occurrence for AFB_1_ in feeds and AFM_1_ in milk was modelled as beta distribution. 

#### 5.3.2. Exposure Assessment

AFM_1_ exposure was determined as a product of milk consumed per day and the concentration of AFM_1_ in milk divided by individual body weights (Equations (1) and (2)). Information on milk consumption for children five years and below, children between six and eighteen years old, and adults was collected using 24-hour and 7-day dietary recall. Body weights assumptions included: 60 kg (standard deviation (SD) of 5) for adult males, 55 kg for adult women (SD of 4), a range of 25–50 kg for children aged 6–18 years, and a range of 5 to 25 kg for children less than 5 years of age. Adults’ body weights were modelled as a normal distribution, whereas children’s weight was modelled as a uniform distribution due to their high variability. The distribution of human aflatoxin exposure was simulated using the Monte Carlo statistical method that involves the random sampling of each probability distribution within the model to produce hundreds or thousands of scenarios or iterations [15].

(1) $$AFM1\left(\mu g\right)=Milk\frac{consumption}{day\left(L\right)}\;x\;Concentration\left(\frac{\mu g}{L}\right)$$

(2) $$AFM1\;intake\;per\;kg\;body\;weight=\frac{AFM1\left(\mu g\right)}{Body\;weight\left(kg\right)}$$

#### 5.3.3. Consequence Assessment

Consequence assessment describes the relationship between specified exposures to AFM_1_ and adverse health consequences based on cancer potency established by the Joint FAO/WHO Expert Committee on Food Additives. Cancer potency is an increase in annual HCC or primary liver cancer incidence rate per unit change in aflatoxin exposure which varies across populations by hepatitis B virus (HBV) status. In hepatitis B surface-antigen positive (HBsAg+) individuals, potency has been estimated to be 0.3 cancers per year per 100,000 population per ng AFB_1_ per kg body weight per day. In hepatitis B surface-antigen negative (HBsAg−) individuals, the potency was 0.01 cancers per year per 100,000 population per ng AFB_1_ per kg body weight per day (Shephard, 2008). The HBsAg+ prevalence rate in Kenya was assumed to be 13% based on an estimate range of 11% to 15% in Kenya [24]. 

#### 5.3.4. Risk Estimation

Risk estimation was done by integrating results from release, exposure and consequence assessments. The annual incidence rate (expressed as cancers per year per 100,000 population) for HCC from AFM_1_ exposure was obtained as the product of the exposure data and an average carcinogenic potency (Equation 3) [18]. A Monte Carlo simulation was performed with 5000 iterations to come up with possible distributions of risk. On each iteration, the @RISK software sampled values from each probability distribution and combined them according to the Excel model.

(3) Probability of cancer per 100,000 population=AFM1 intake per kg body weight x Dose response

## Figures and Tables

**Figure 2:**
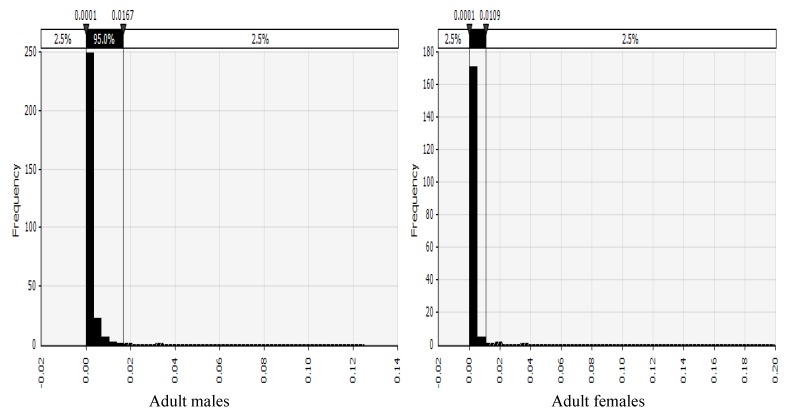
Probability distribution of risk of cancer in adult males and females from a semi-arid AEZ in Kenya.

**Figure 3:**
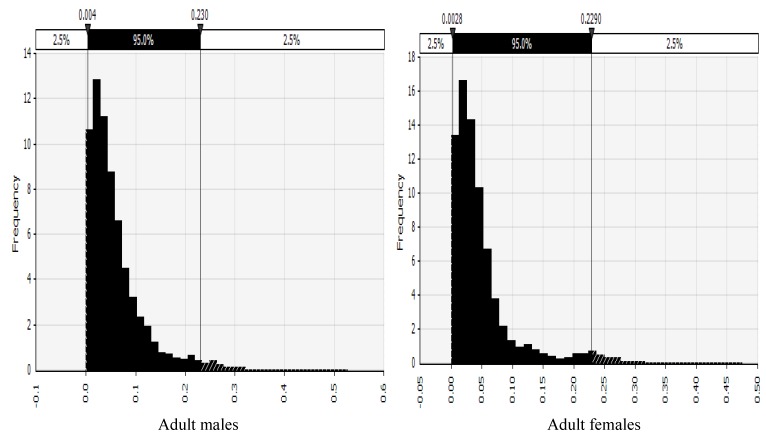
Probability distribution of risk of cancer in adult males and females from a sub-humid AEZ in Kenya.

**Figure 4:**
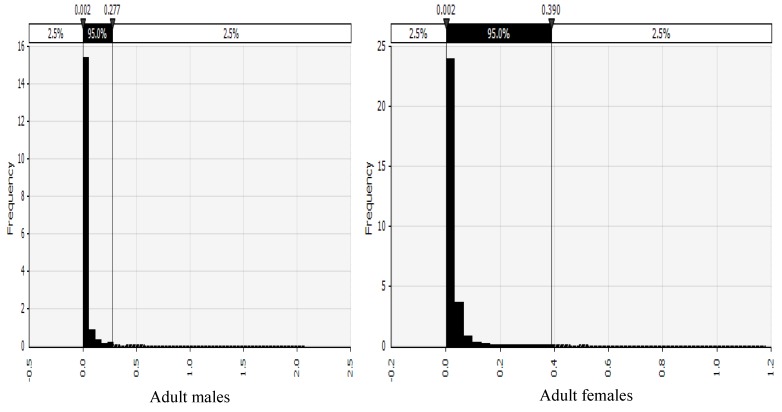
Probability distribution of risk of cancer in adult males and females from a humid AEZ in Kenya.

**Figure 5:**
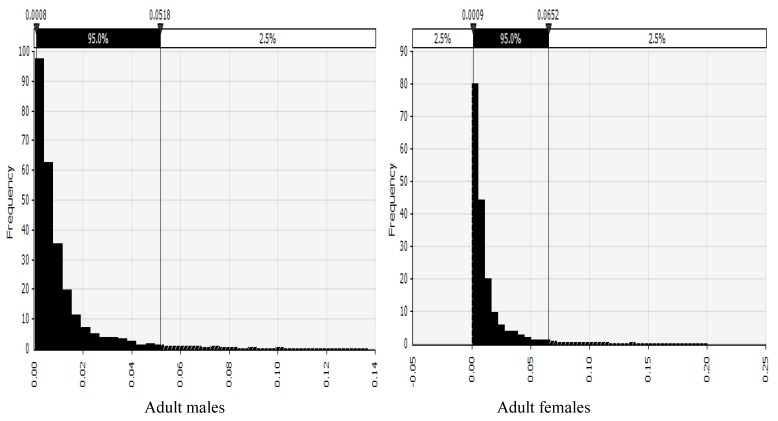
Probability distribution of risk of cancer in adult males and females from a temperate AEZ in Kenya.

**Table 1:** Mean aflatoxin M_1_ (AFM_1_) levels in milk samples from cows fed with or without concentrates/maize-based feeds.

Agro-Ecological Zone	Mean AFM_I_ Levels in Milk from Cows Fed with Concentrates or Maize Based Feeds	Probability of Samples Exceeding EU Limits (50 ng/kg)	Mean AFM_I_ Levels in Milk from Cows Not Fed Concentrates or Maize Based Feeds	Probability of Samples Exceeding 50 ng/kg
**Semi-Arid**	n/a	-	8.3 (*n* = 53)	0.04
**Sub-Humid**	370.7 (*n* = 2)	*	4.7 (*n* = 30)	**
**Humid**	52.9 (*n* = 67)	0.46	10 (*n* = 21)	**
**Temperate**	34.6 (*n* = 47)	0.13	21.3 (*n* = 41)	0.08

* All samples were above 0.05 ng/g; ** all samples were below 0.05 ng/g.

**Table 2:** Summary statistics of cow milk consumption in liters per day across agro–ecological zones (AEZs) in Kenya.

AEZ	Mean	Median
**Semi-Arid (*n* = 200)**	0.2	0.2
**Sub-Humid (*n* = 112)**	0.3	0.2
**Humid (*n* = 192)**	0.3	0.3
**Temperate (*n* = 416)**	0.5	0.4
**Total (*n* = 920)**	0.4	0.3

**Table 3:** Estimated annual hepatocellular carcinoma (HCC) incidence rate per 100,000 among different sub-populations (95% confidence intervals).

Agro-Ecological Zone	Adult Male	Adult Female	Child 6–18 Years	Child <5 Years
Semi-arid	4 × 10^−5^ (3 × 10^−5^–5 × 10^−5^)	5.9 × 10^−3^ (4.2 × 10^−3^–7.5 × 10^−3^)	2 × 10^−5^ (2 × 10^−5^–3 × 10^−5^)	1 × 10^−4^ (8 × 10^−5^–1 × 10^−4^)
Sub-humid	3.2 × 10^−3^ (2.3 × 10^−3^–4 × 10^−3^)	1.7 × 10^−3^ (1.2 × 10^−3^–2.1 × 10^−3^)	5 × 10^−6^ (4 × 10^−6^–6 × 10^−6^)	1.3 × 10^−2^ (9.2 × 10^−3^–1.7 × 10^−2^)
Humid	3.3 × 10^−3^ (2.3 × 10^−3^–4.2 × 10^−3^)	2 × 10^−4^ (1 × 10^−4^–3 × 10^−4^)	2.7 × 10^−3^ (1.9 × 10^−3^–3.4 × 10^−3^)	2.3 × 10^−3^ (1.6 × 10^−4^–2.9 × 10^−3^)
Temperate	1.3 × 10^−3^ (9 × 10^−4^–1.7 × 10^−3^)	3 × 10^−4^(2 × 10^−4^–4 × 10^−4^)	7 × 10^−4^ (5 × 10^−4^–9 × 10^−4^)	2.4 × 10^−3^ (1.7 × 10^−3^–3 × 10^−3^)
All	2.9 × 10^−3^ (95% CI: 2.5 × 10^−3^–3.3 × 10^−3^)	3.5 × 10^−3^ (95% CI: 3 × 10^−3^–3.9 × 10^−3^)	1.4 × 10^−3^ (95% CI: 1.2 × 10^−3^–1.5 × 10^−3^)	2.7 × 10^−3^ (95% CI: 2.3 × 10^−3^–3 × 10^−3^)

**Table 4:** Parameters used in the risk model.

Risk Assessment Step	Name	Distributions
Release assessment	AFM_1_ occurrence in milk in extensive rearing without concentrates	Risk Beta (Number of positive + 1, Number of sample–Number positive + 1)
Release assessment	AFM_1_ occurrence in milk in intensive rearing without concentrates	Risk Beta (Number of positive + 1, Number of sample–Number positive + 1)
Release assessment	AFB_1_ occurrence in feed in extensive rearing with concentrates	Risk Beta (Number of positive + 1, Number of sample–Number positive + 1)
Release assessment	AFB_1_ occurrence in feed in intensive rearing with concentrates	Risk Beta (Number of positive + 1, Number of sample–Number positive + 1)
Release assessment	AFM_1_ occurrence in milk in extensive rearing with concentrates	Risk Beta (Number of positive + 1, Number of sample–Number positive + 1)
Release assessment	AFM_1_ occurrence in milk in intensive rearing with concentrates	Risk Beta (Number of positive + 1, Number of sample–Number positive + 1)
Exposure assessment	Frequency of milk consumption (rate)	Risk Duniform (bootstrap of raw data)
Exposure assessment	Whether milk was consumed that day	Risk Binomial (1, rate)
Exposure assessment	Volume of milk consumed, if consumed	Risk Duniform (bootstrap of raw data)
Exposure assessment	AFM_1_ status in milk	Risk Binomial (1, occurrence of AFM_1_)
Exposure assessment	AFM_1_ levels in milk	Risk Duniform (bootstrap of raw data)
Exposure assessment	Body weight	Risk Normal
Exposure assessment	Hepatitis B prevalence	Risk Binomial (1, hepatitis B prevalence)

## Supplementary Materials

The following are available online at https://www.mdpi.com/2072-6651/11/8/469/s1, Figure S1: Probability distribution of risk of cancer in adult males from a semi-arid AEZ. Figure S2: Probability distribution of risk of cancer in adult females from a semi-arid AEZ. Figure S3: Probability distribution of risk of cancer in children 6–18 years old from a semi-arid AEZ. Figure S4: Probability distribution of risk of cancer in children less than five years old from a semi-arid AEZ. Figure S5: Probability distribution of risk of cancer in adult males from a sub-humid AEZ. Figure S6: Probability distribution of risk of cancer in adult females from a sub-humid AEZ. Figure S7: Probability distribution of risk of cancer in children 6–18 years old from a sub-humid AEZ. Figure S8: Probability distribution of risk of cancer in children less than five years old from a sub-humid AEZ. Figure S9: Probability distribution of risk of cancer in adult males from a humid AEZ. Figure S10: Probability distribution of risk of cancer in adult females from a humid AEZ. Figure S11: Probability distribution of risk of cancer in children 6–18 years old from a humid AEZ. Figure S12: Probability distribution of risk of cancer in children less than five years old from a humid AEZ. Figure S13: Probability distribution of risk of cancer in adult males from a temperate AEZ. Figure S14: Probability distribution of risk of cancer in adult females from a temperate AEZ. Figure S15: Probability distribution of risk of cancer in children 6–18 years old from a temperate AEZ. Figure S16: Probability distribution of risk of cancer in less than five years old from temperate AEZ.
