# Supplementary material for: Aflatoxin Exposure from Milk in Rural Kenya and the Contribution to the Risk of Liver Cancer
Source: Toxins (Basel). 2019 Aug 10;11(8):469. doi: 10.3390/toxins11080469 (PMC6722829; doi:10.3390/toxins11080469)
Supplement: Supplementary file 1 [file toxins-11-00469-s001.pdf]

Toxins **2019**, *11*, 469; doi:10.3390/toxins11080469

Supplementary Materials: Aflatoxin Exposure from Milk in Rural Kenya and the Contribution to the Risk of Liver Cancer

Anima J. Sirma, Kohei Makita, Delia Grace Randolph, Daniel Senerwa and Johanna F. Lindahl

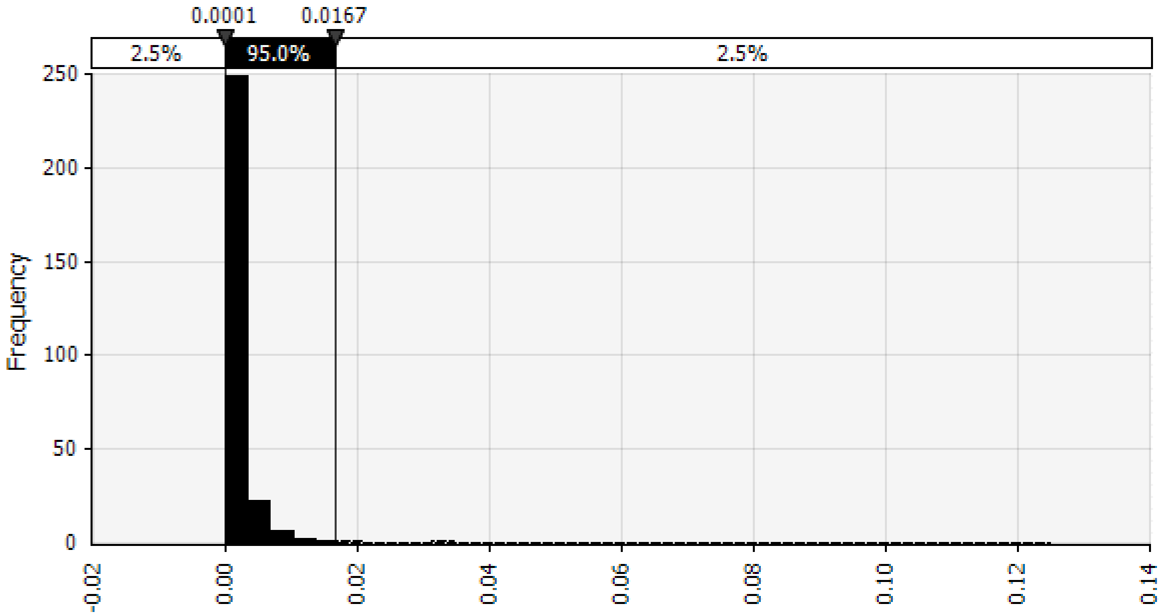

Figure S1. Probability distribution of risk of cancer in adult males from a semi-arid agro–ecological zone in Kenya.

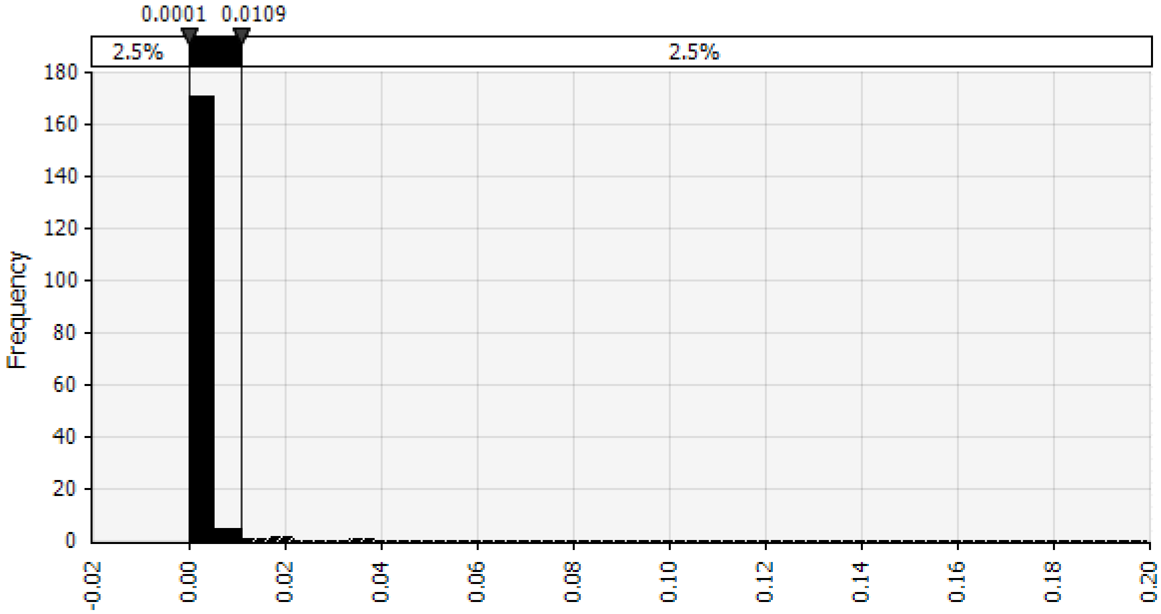

Figure S2. Probability distribution of risk of cancer in adult females from a semi-arid agro–ecological zone in Kenya.

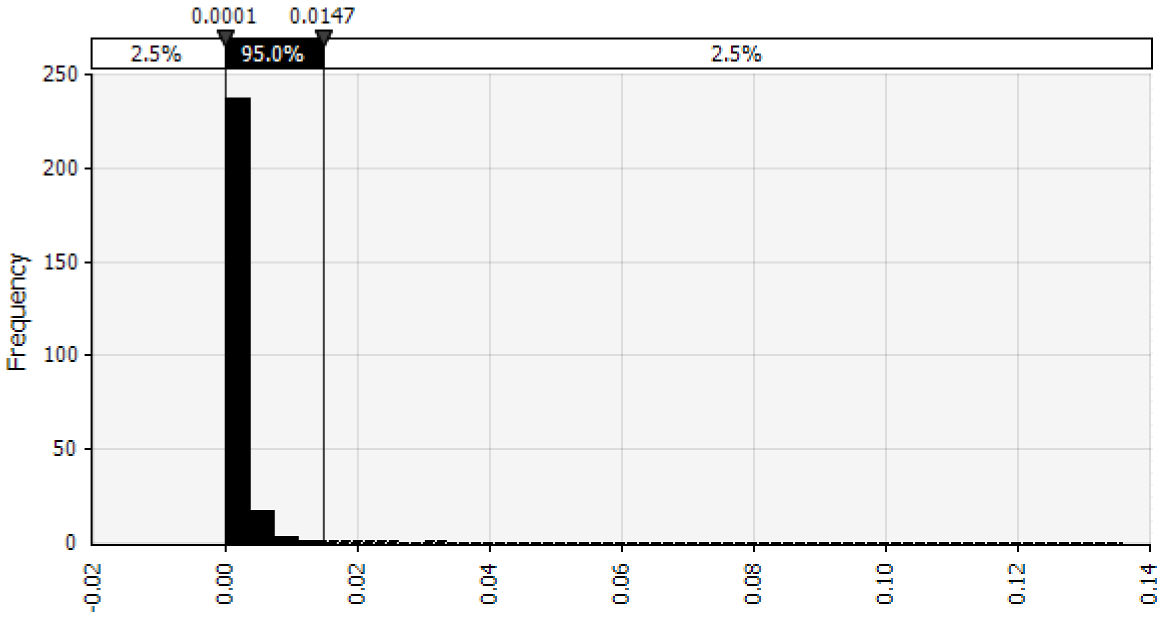

Figure S3. Probability distribution of risk of cancer in children 6–18 years old from a semi-arid agro–ecological zone in Kenya.

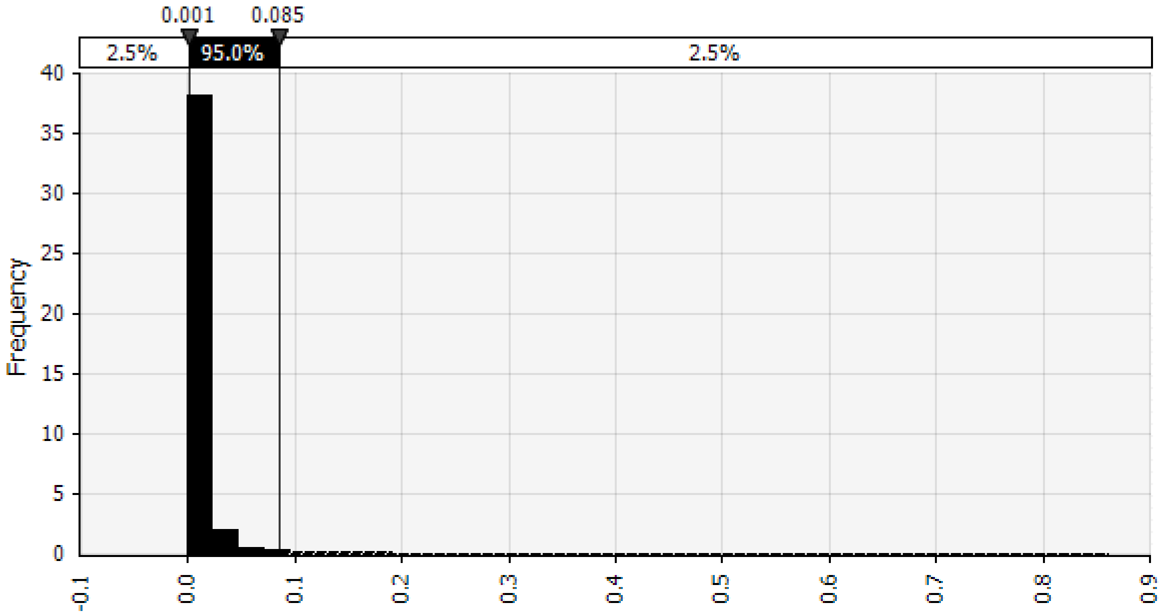

Figure S4. Probability distribution of risk of cancer in children less than five years old from a semi-arid agro–ecological zone in Kenya.

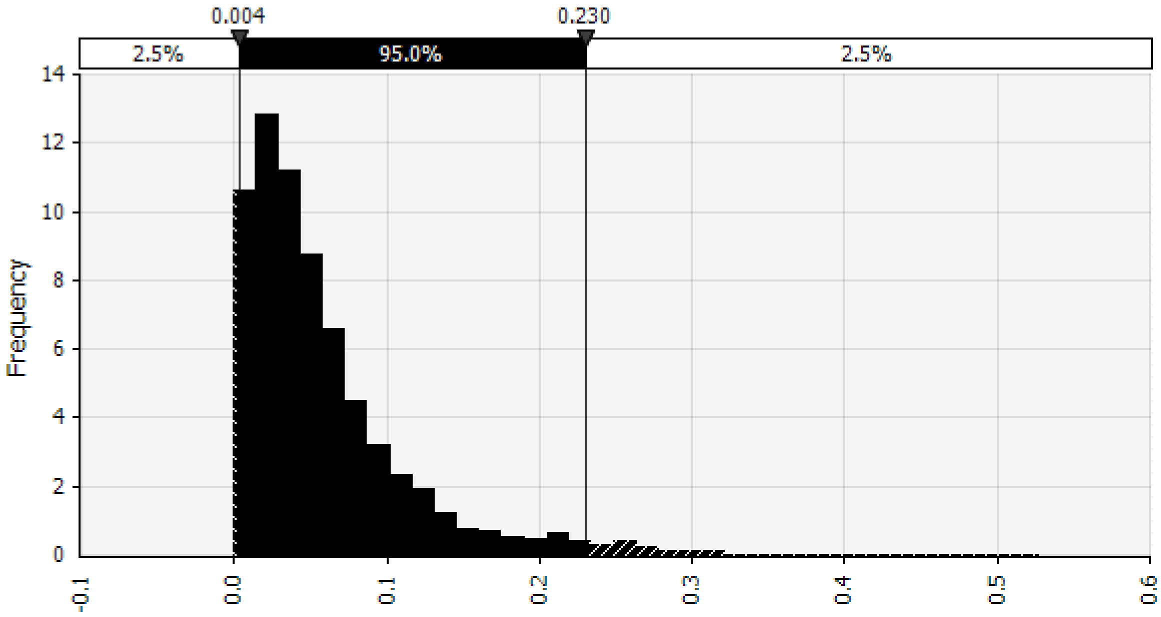

Figure S5. Probability distribution of risk of cancer in adult males from a sub-humid agro–ecological zone in Kenya.

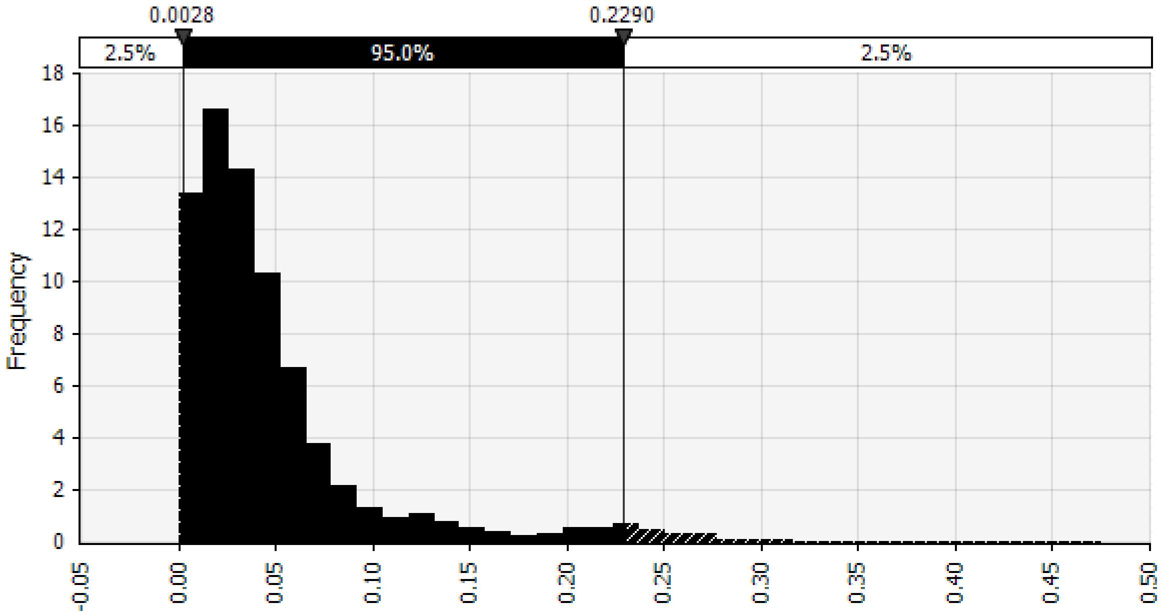

Figure S6. Probability distribution of risk of cancer in adult females from a sub-humid agro–ecological zone in Kenya.

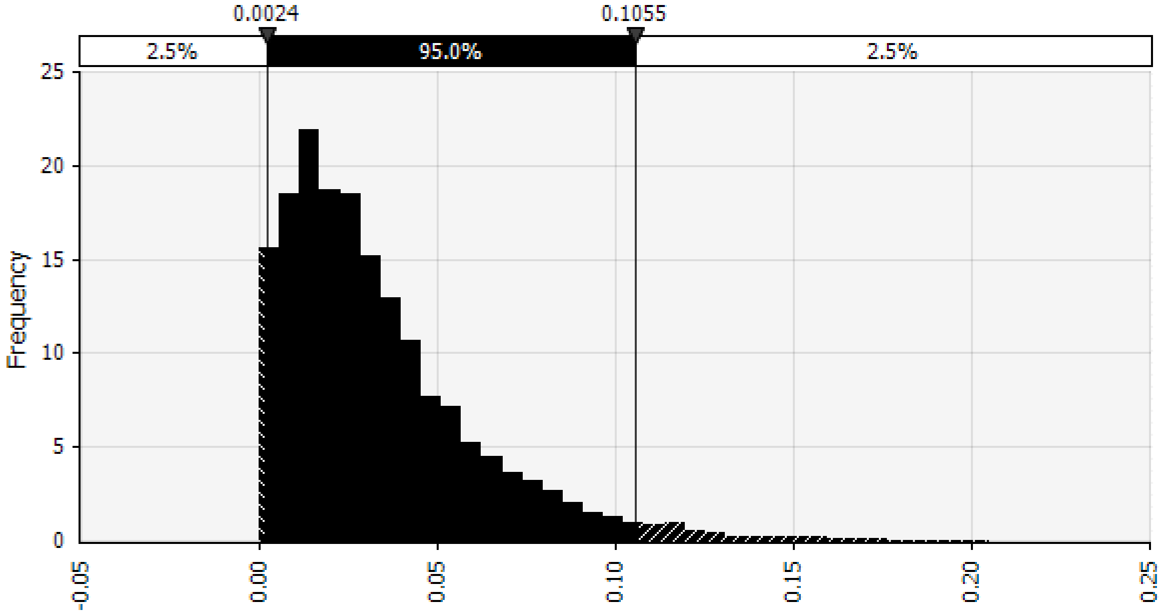

Figure S7. Probability distribution of risk of cancer in children 6–18 years old from a sub-humid agro–ecological zone in Kenya.

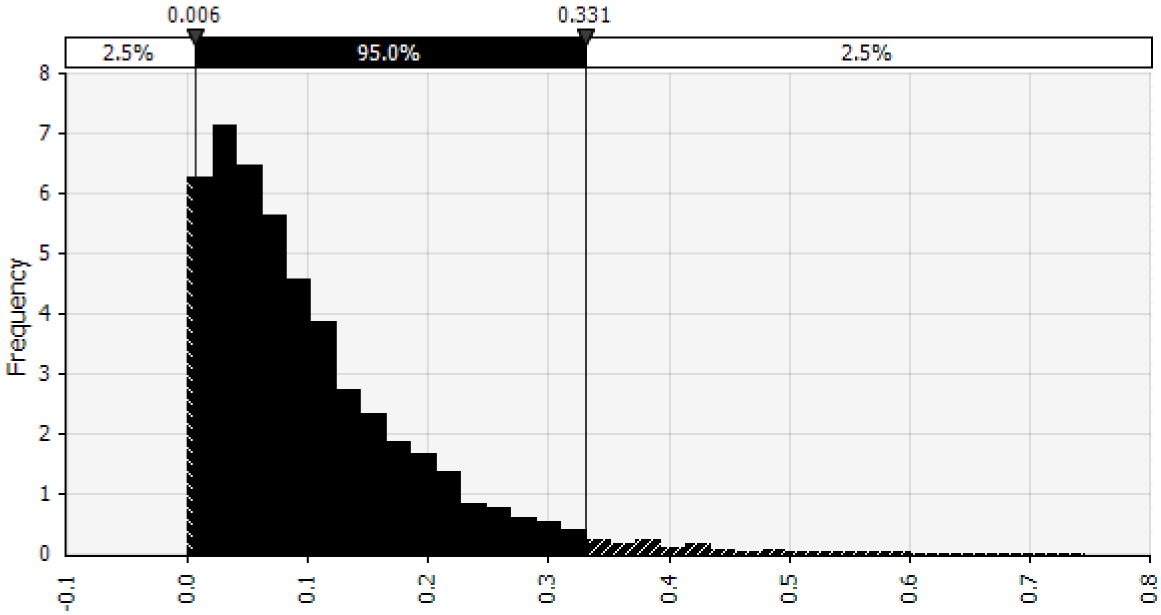

Figure S8. Probability distribution of risk of cancer in children less than five years old from a sub-humid agro–ecological zone in Kenya.

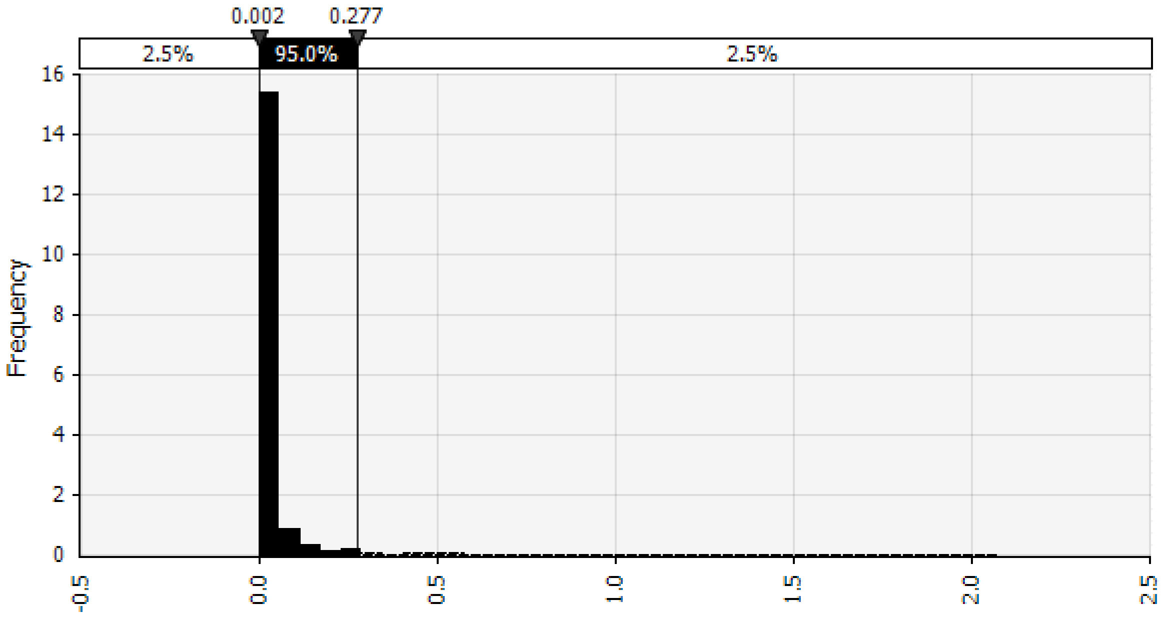

Figure S9. Probability distribution of risk of cancer in adult males from a humid agro–ecological zone in Kenya.

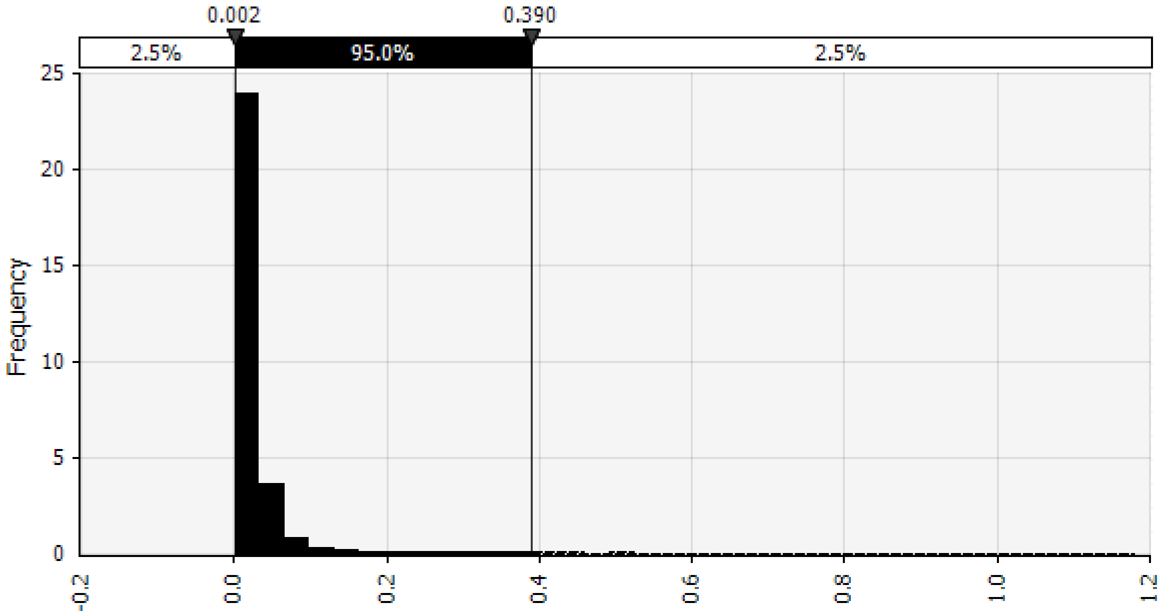

Figure S10. Probability distribution of risk of cancer in adult females from a humid agro–ecological zone in Kenya.

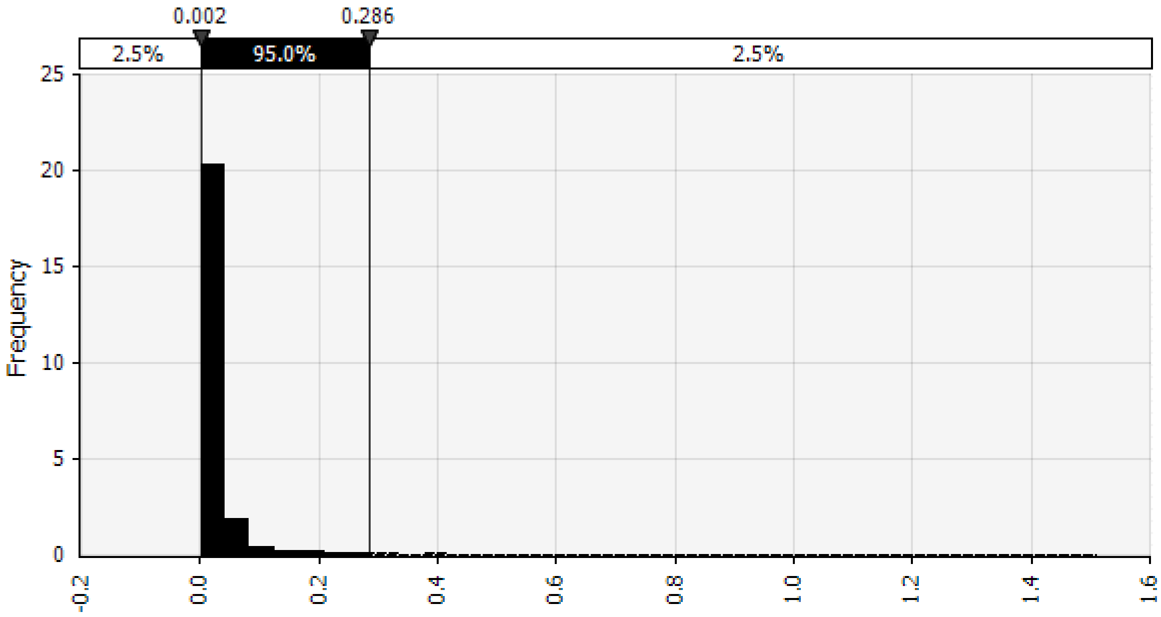

Figure S11. Probability distribution of risk of cancer in children 6–18 years old from a humid agro–ecological zone in Kenya.

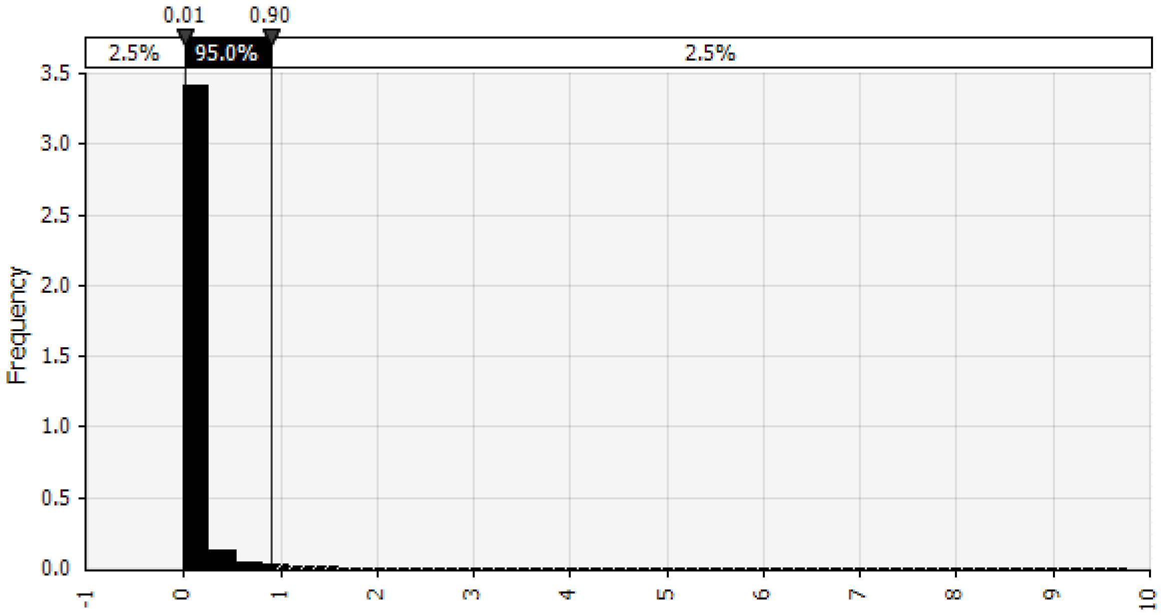

Figure S12. Probability distribution of risk of cancer in children less than five years old from a humid agro–ecological zone in Kenya.

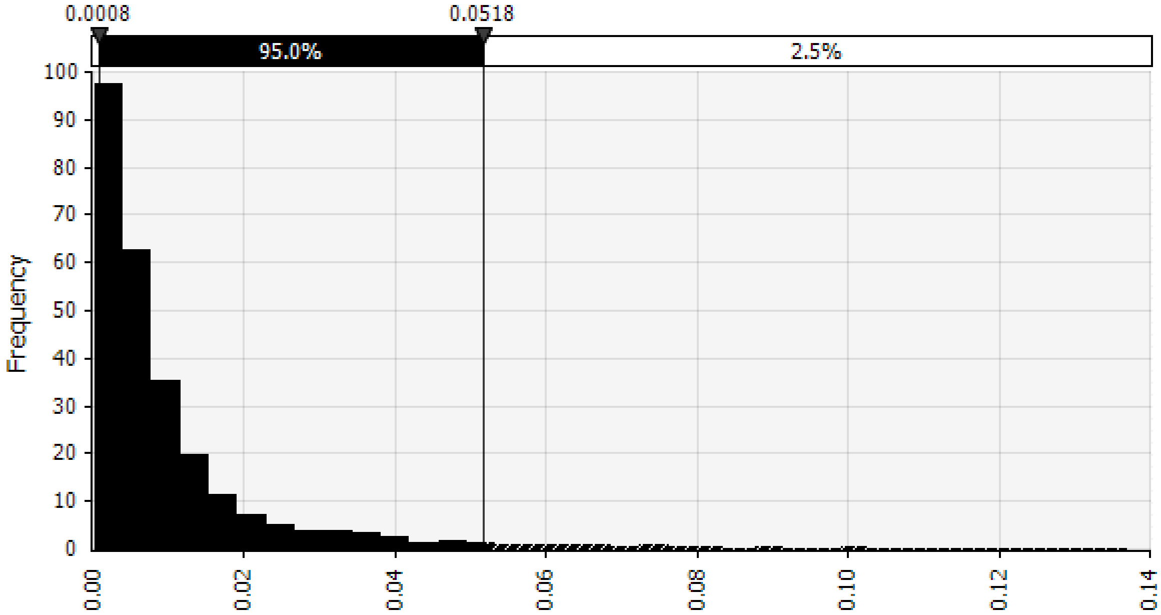

Figure S13. Probability distribution of risk of cancer in adult males from a temperate agro–ecological zone in Kenya.

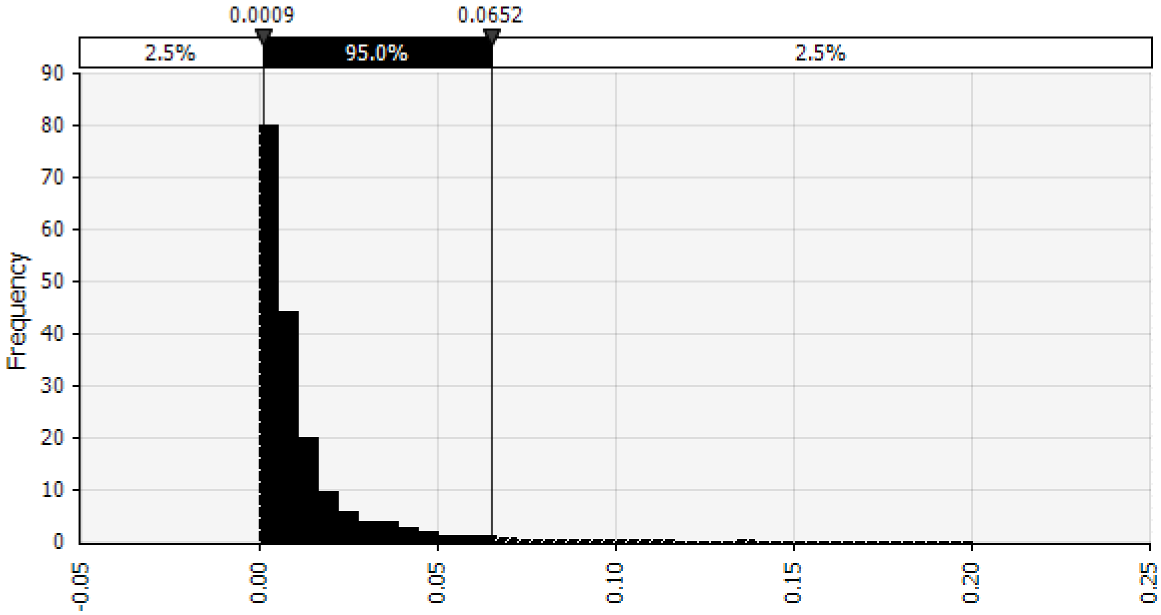

Figure S14. Probability distribution of risk of cancer in adult females from a temperate agro–ecological zone in Kenya.

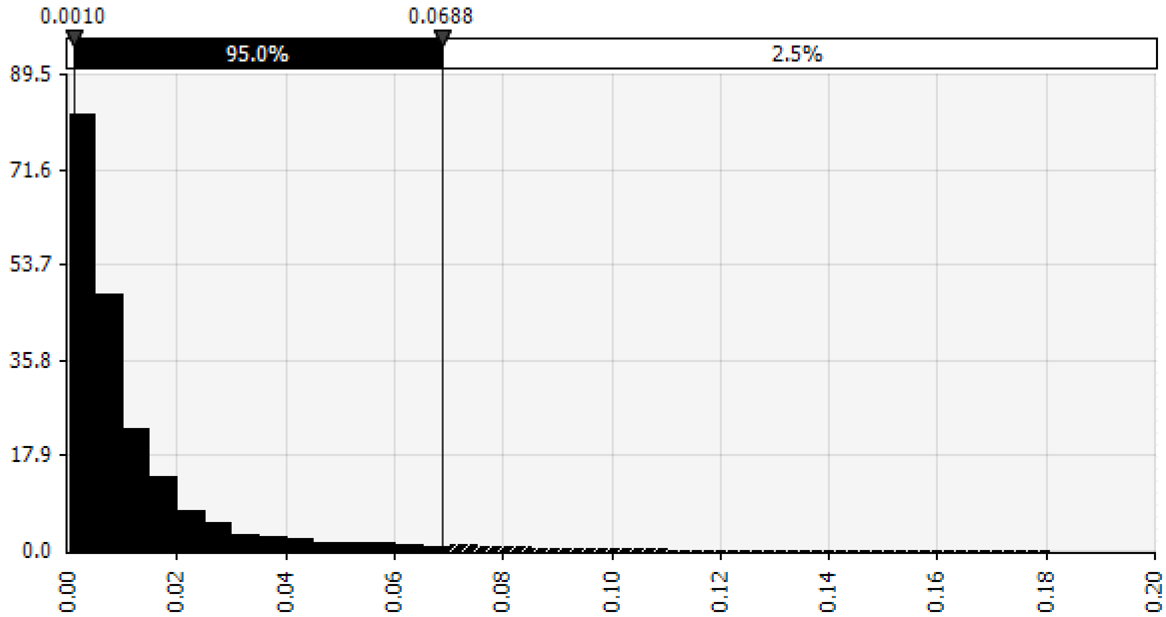

Figure S15. Probability distribution of risk of cancer in children 6–18 years old from a temperate agro–ecological zone in Kenya.

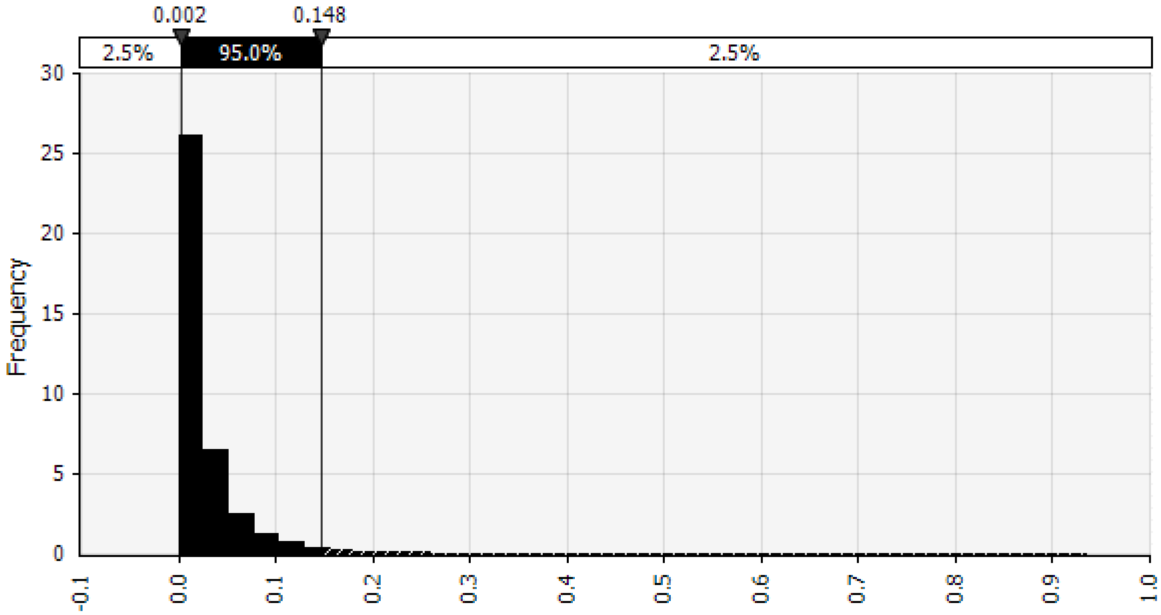

Figure S16. Probability distribution of risk of cancer in less than five years old from a temperate agro–ecological zone in Kenya.
